# KARIs, Ghrelin Receptor Agonists With Excellent Brain Permeability, Increase Food Intake and Attenuate the Muscle Loss in Mice

**DOI:** 10.1002/jcsm.70277

**Published:** 2026-04-01

**Authors:** Hee Ji Yoon, Wan Hui Han, Sang Bo Kim, Won Gyeong Lee, Seo Young Choi, Byung Jo Choi, Minyeong Pang, Min‐Koo Choi, Jihoon Lee, Im‐Sook Song, Min Hee Park, Hee Kyung Jin, Jae‐sung Bae

**Affiliations:** ^1^ KNU AD Research Institute Kyungpook National University Daegu Republic of Korea; ^2^ Department of Physiology, Cell and Matrix Research Institute, School of Medicine Kyungpook National University Daegu Republic of Korea; ^3^ College of Pharmacy Dankook University Cheonan Republic of Korea; ^4^ BK21 FOUR Community‐Based Intelligent Novel Drug Discovery Education Unit, Vessel‐Organ Interaction Research Center (VOICE), College of Pharmacy and Research Institute of Pharmaceutical Sciences Kyungpook National University Daegu Republic of Korea; ^5^ Department of Laboratory Animal Medicine, College of Veterinary Medicine Kyungpook National University Daegu Republic of Korea

**Keywords:** brain permeability, food intake, ghrelin receptor agonist, muscle growth, small molecule compound

## Abstract

**Background:**

Ghrelin regulates appetite, gastrointestinal motility and growth hormone (GH) secretion through activation of the growth hormone secretagogue receptor (GHSR‐1a) in hypothalamic neurons, with downstream effects on adipose tissue and skeletal muscle. Although ghrelin and synthetic GHSR‐1a agonists have been investigated for the treatment of anorexia, sarcopenia and cancer cachexia, their clinical utility has been limited by unfavourable pharmacokinetics. We recently discovered a novel class of small‐molecule compounds, termed KARIs, which act as potent GHSR‐1a agonists with excellent brain permeability. Here, we evaluated their efficacy as GHSR‐1a agonists in vitro and in vivo.

**Method:**

GHSR‐1a agonistic effects of KARIs were assessed using calcium influx and competitive binding assays in human GHSR‐1a‐expressing cells, and molecular docking simulations were conducted. Pharmacokinetic properties of KARIs (1 mg/kg i.v, 10 mg/kg p.o) were compared with anamorelin (3 mg/kg i.v., 30 mg/kg p.o.). In vivo efficacy was evaluated in mouse models of postoperative ileus (POI; 10, 20 or 30 mg/kg), age‐related sarcopenia (10 mg/kg/day for 4 weeks) and cancer cachexia (10 or 30 mg/kg/day from Days 9 to 24 after CT26 tumour induction).

**Results:**

KARIs directly interacted with key GHSR‐1a residues (Phe279) and increased calcium influx (EC_50_: KARI 101 = 1.83 ± 1.05 μM; KARI 201 = 3.36 ± 1.09 μM). KARIs showed higher oral bioavailability (BA: KARI 101 = 63%; KARI 201 = 68%) and brain distribution (KARI 101 = 2.2; KARI 201 = 3.7) than anamorelin (BA = 45%, brain distribution = 0.1). KARIs significantly activated hypothalamic neurons (KARI 101 *p* = 0.029, KARI 201 *p* = 0.0152), resulting in increased food intake and improved gastric emptying and colonic transit in POI mice. In aged mice, KARIs markedly elevated plasma GH levels and restored gastrocnemius muscle mass (KARI 101 *p* = 0.0018, KARI 201 *p* = 0.0006) and improved rota‐rod performance (*p* < 0.0001) by downregulating expression of atrophic genes and upregulating myogenic genes. In cancer cachexia mice, KARIs improved food intake and preserved skeletal muscle mass at lower doses (gastrocnemius, 10 mg/kg; *p* < 0.0001) than synthetic GHSR ‐ 1a agonist (anamorelin, 30 mg/kg) and enhanced functional recovery (rota‐rod test, 10 mg/kg; KARI 101 *p* = 0.045, KARI 201 *p* = 0.003) without affecting tumour growth.

**Conclusions:**

KARIs are potent, brain‐penetrant GHSR‐1a agonists with favourable pharmacokinetics compared to anamorelin. They enhance appetite, preserve skeletal muscle mass and improve physical performance in models of aging and cancer cachexia, supporting their potential as next‐generation therapies for anorexia and muscle wasting.

## 1 Introduction

Ghrelin, an orexigenic hormone mainly secreted by the stomach, plays a critical role in regulating energy homeostasis and growth [1, 2]. Its biological effects are mediated through binding to the growth hormone secretagogue receptor (GHSR‐1a), which is abundantly expressed in hypothalamic neurons [2, 3]. Activation of these neurons through ghrelin/GHSR‐1a signalling stimulates appetite and enhances gastrointestinal motility via the vagus nerve, thereby promoting increased food intake [1, 2, 3, 4]. In addition, this signalling induces the release of growth hormone (GH) from the anterior pituitary gland [2, 4]. Through GH secretion and subsequent activation of downstream pathways, ghrelin influences multiple peripheral organs, particularly adipose tissue and skeletal muscle, contributing to fat deposition, muscle growth and overall metabolic adaptation [4, 5].

Owing to these multifaceted actions, ghrelin has been extensively studied as a potential therapeutic target for the management of anorexia, impaired intestinal motility, weight loss, sarcopenia, and cancer cachexia [5, 6]. Indeed, some studies have demonstrated that exogenous ghrelin administration transiently increases food intake, improves gastrointestinal transit and exerts anabolic effects on lean body mass [7, 8, 9]. However, ghrelin itself has been limited due to its short half‐life, rapid degradation by plasma enzymes and unfavourable pharmacokinetic properties, which necessitate frequent administration to maintain efficacy [10, 11]. Moreover, as a peptide hormone, ghrelin requires parenteral delivery, further limiting its practicality for long‐term therapeutic use [10].

To address these challenges, several small‐molecule GHSR‐1a agonists have been developed, including anamorelin, which is approved in Japan for the treatment of cancer cachexia. Although anamorelin has demonstrated efficacy in improving appetite and body weight, it has not been approved by the US Food and Drug Administration, largely due to its limited efficacy in improving muscle strength and physical function. These limitations underscore the need for next‐generation GHSR‐1a agonists with improved efficacy in preserving skeletal muscle mass and function. Accordingly, targeting the GHSR‐1a, especially those abundantly expressed in the brain and directly involved in the regulation of appetite and GH release, has emerged as a more promising strategy, and many researchers have made considerable efforts to develop GHSR‐1a agonists [12, 13, 14].

In our previous study, we identified a novel class of small‐molecule compounds, termed KARIs, which were originally developed as potential therapeutics for Alzheimer's disease by targeting sphingolipid enzyme but were unexpectedly found to also act as potent agonists of GHSR‐1a [15]. These compounds exhibited favourable pharmacokinetic properties, including excellent bioavailability and high brain distribution [15]. Based on these findings, the present study aimed to further characterize the role of KARIs as ghrelin receptor agonists. We demonstrated that KARIs effectively stimulated food intake, enhanced gastrointestinal transit and attenuated muscle loss in various in vivo mouse models, including postoperative ileus (POI), age‐related sarcopenia and cancer cachexia. Collectively, our results highlight that brain‐penetrant ghrelin receptor agonists, KARIs, have significant therapeutic potential for the treatment of conditions associated with anorexia and muscle loss, including sarcopenia and cancer cachexia.

## 2 Materials and Methods

### 2.1 Chemicals

KARI 101 (2‐amino‐2‐(1‐decyl‐1H‐1,2,3‐triazol‐4‐yl)propane‐1,3‐diol) and KARI 201 (2‐amino‐2‐(1‐nonyl‐1H‐1,2,3‐triazol‐4‐yl)propane‐1,3‐diol) were synthesized as previously described [15]. The structure and details of these small compounds are shown in Figure S1.

### 2.2 Human Ghrelin/GHSR‐1α Cellular Functional Assay

The GHSR‐1α agonist effect of KARIs was performed at Eurofins Pharma Discovery (France, Study number: 100057175).

### 2.3 Pharmacokinetics and Brain Distribution

C57BL/6 mice were administered KARI 101 or KARI 201 at doses of 1 mg·kg^−1^ (i.v.) or 10 mg·kg^−1^ (p.o.) and anamorelin at doses of 3 mg·kg^−1^ (i.v.) or 30 mg·kg^−1^ (p.o.). Venous blood samples were collected at 15 and 30 min and 1‐, 2‐, 4‐, 8‐, 24‐ and 48‐h postdose. About 30 μL of plasma was separated from the whole blood by centrifugation and stored at −80°C until analysis. Brain samples were also collected at same time point postdoses thoroughly rinsed with physiological saline and weighed. About 20% tissue homogenates were prepared by homogenizing the tissue samples with four volumes of saline. A 30 μL aliquot of plasma or a 50 μL aliquot of tissue homogenates was mixed with 30 μL aliquot of [^13^C] caffeine aqueous solution (100 ng/mL), used as an internal standard (IS), and 500 μL of ethyl acetate. The mixture was vigorously vortexed for 5 min and then centrifuged at 13 200 × g for 5 min. The 450 μL supernatant was transferred to a clean tube and evaporated it to dryness using a Speed Vac (60°C, 4 mbar, 25 min). The residue was reconstituted with 120 μL of 85% acetonitrile, vortexed for 5 min and centrifuged at 16000 × g for 5 min. Then, the 120 μL supernatant was transferred to a autosampler vial and a 5 μL aliquot of the supernatant was injected into an Agilent 6470 LC–MS/MS system. Separation was performed on a Synergi Polar RP column (150 × 2.0 mm, 4 μm; Phenomenex, Torrance, CA, USA) using a mobile phase that consisted of acetonitrile and water (90:10, v/v) with 0.1% formic acid at a flow rate of 0.2 mL min^−1^. Quantification was carried out using multiple reaction monitoring (MRM) at m/z 327 → 310 for KARI 101, 285 → 268 for KARI 201, 547 → 276 for anamorelin, and m/z 198 → 140 for [^13^C] caffeine (IS) in positive ionization mode and collision energy of 15–20 eV. The pharmacokinetic parameters such as maximum concentration (C_max_) and the time to reach C_max_ (T_max_), the area under the plasma concentration–time curve during the period of observation (AUC_last_), AUC to infinite time (AUC_∞_), total clearance (CL), volume of distribution (Vd), the terminal half‐life (T_1/2_) and mean residence time (MRT) of KARI 101, KARI 201 or anamorelin were calculated using a noncompartment analysis (WinNonlin 5.1; Pharsight, Cary, NC, USA). Bioavailability (%) values were calculated by dividing AUCp.o. normalized by p.o. dose/AUCi.v. normalized by i.v. dose × 100.

### 2.4 Chemical Administration

To examine the effects of KARI 101, KARI 201 and anamorelin as GHSR‐1α agonists in vivo, 3‐ or 23‐month‐old C57BL/6 mice (The Jackson Laboratory) were used. Both male and female mice were used for all experiments. Mice were housed at a 12‐h day/12‐h night cycle, 21°C–22°C and 50%–60% humidity with free access to water and food pellets before compounds administration. KARI compounds (10 mg·kg^−1^ for 2 or 4 weeks) or anamorelin (30 mg·kg^−1^ for 4 weeks) were administered daily p.o., and food intake or body weight was measured at the same time every day during the administration period. The rota‐rod test was performed weekly throughout the treatment period, whereas the grip strength test was conducted during the final week before sacrifice. After behavioural tests, mice were anaesthetised by an intraperitoneal (IP) injection of a mixture of ketamine (100 mg·kg^−1^) and xylazine (10 mg·kg^−1^), and blood was collected into sodium heparin‐coated tubes via intracardial bleed. After blood collection, mice were transcardially perfused with PBS. Fats (subcutaneous and visceral) and hindlimb muscles (quadriceps, gastrocnemius and tibialis anterior) were dissected, weighed and snap‐frozen for further analysis. All animal experiments were performed in accordance with protocols approved by the Institutional Animal Care and Use Committee (IACUC) at Kyungpook National University.

### 2.5 Postoperative Ileus (POI) Mouse Model

Prior to the experiments, mice were fasted for 20–22 h with free access to water. POI was induced through a previously described surgical procedure [16, 17]. Briefly, mice were intraperitoneally anaesthetised by a mixture of ketamine (100 mg·kg^−1^) and xylazine (10 mg·kg^−1^). Surgery was performed under sterile conditions. Mice underwent control surgery of only laparotomy or of laparotomy followed by intestinal manipulation. A midline abdominal incision was made, and the peritoneum was opened over the linea alba. The small bowel was carefully exteriorized, layered on a sterile moist gauze pad and manipulated from the distal duodenum to the cecum for 5 min by using sterile moist cotton applicators. Contact or stretch on the stomach or colon was strictly avoided. At the end of surgery, 0.2 mL of dye (2.5% trypan blue in saline) was carefully injected into the proximal colon (1 cm distal to the cecum) using a hypodermic needle for colonic transit analysis. After the surgical procedure, the abdomen was closed by a continuous two‐layer suture, and mice were allowed to recover in a heated (32°C) recovery cage.

### 2.6 Cancer Cachexia Mouse Model

The CT26 tumour model is frequently used for the study of cancer cachexia and has been previously described [18]. Colon‐26 tumour cells (Korean cell line bank) were cultured under standard conditions in RPMI 1640 supplemented with 10% foetal bovine serum and 1% penicillin–streptomycin (all from Gibco). Confluent CT26 Petri dishes (70%–80%) were trypsinated and then washed repeatedly with RPMI 1640 to harvest the cells. After confirming viability (≥ 80%) of the cells with trypan blue, 10^6^ cells were inoculated in 200 μL of PBS subcutaneously into the right flank of 8‐week‐old BALB/c mice. Nine days after inoculation, tumours were palpable (about 5 mm in diameter) in mice, and then mice were randomly divided for daily administration of 10 or 30 mg·kg^−1^ KARI 101, KARI 201 or 30 mg·kg^−1^ anamorelin via oral gavage until the end of the experiment at day 24. Food intake, body weight and tumour volume were measured every 4 days. Tumour volumes were calculated using the formula: tumour volume (mm^3^) = 0.52 × length × width^2^ in which length and perpendicular width were measured by a vernier calliper. Grip strength and rota‐rod tests were performed at Days 22 and 23, respectively. At Day 24, mice were sacrificed, tumours, fats, and hindlimb muscles were removed and weighed, and muscles were used for subsequent analysis.

### 2.7 Behavioural Test

Grip strength in all four limbs (forelimbs and hindlimbs) was measured using a Bio‐GS4 grip strength test meter with a wire mesh grid for four paws. Each mouse was measured nine times consecutively with a 30‐s pause between each measurement. The values were averaged to reduce procedure‐related variability, and the data were presented as averages after normalization against body weight. The rotarod apparatus (accelerating model 47 600; Ugo Basile) was set to an initial speed of 4 rpm, and the acceleration was increased by 32 rpm every 25–30 s. Scores were registered every 2 days, and three independent tests were performed at each measurement. Uniform conditions were carefully maintained for each test, with a rest time of 1 h between trials. Each test was limited to 300 s.

### 2.8 Statistical Analysis

Sample sizes were determined by the G‐Power software (ver 3.1.9.4, with *α* = 0.05 and power of 0.8). In general, statistical methods were not used to recalculate or predetermine sample sizes. Variance was similar within comparable experimental groups. Individuals performing the experiments were blinded to the identity of experimental groups until the end of data collection and analysis for at least one of the independent experiments. All data are representative of at least three independent experiments. In cases where more than two groups were compared to each other, one‐way analysis of variance (ANOVA) was used, followed by Tukey's HSD test. Classical statistical analyses were performed using GraphPad Prism 8.0 software. *p* < 0.05 was considered statistically significant.

## 3 Results

### 3.1 KARIs Induce Intracellular Calcium Influx by Directly Interact With Critical Residue of GHSR‐1a

**FIGURE 1:**
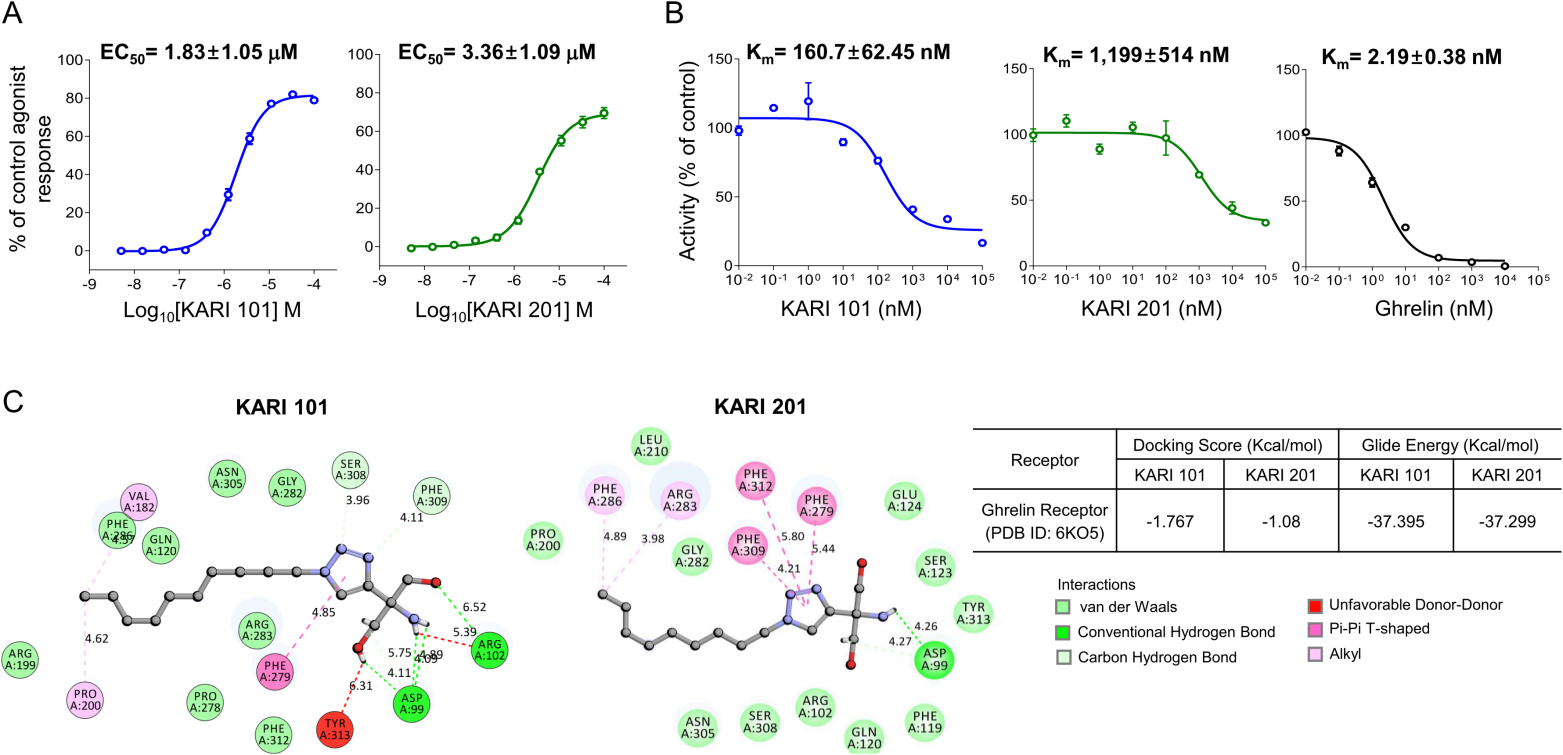
KARIs induce intracellular calcium influx by directly interacting critical residues of GHSR‐1a. (A) Effect of various concentrations of the KARI compounds on intracellular calcium influx in GHSR‐1a stable cells (data are mean ± SEM; *n* = 3 independent experiments). (B) Competition binding of KARI 101, KARI 201 and ghrelin (data are mean ± SEM; *n* = 3 independent experiments). (C) Detailed interaction map through molecular docking simulation between ghrelin receptor (PDB ID:6KO5) and KARI 101 or KARI 201. Error bars represent s.e.m. and may not be visually discernible in some cases due to their small magnitude.

In a previous study, we observed that among small molecule compounds with a 2‐amino‐2‐(1,2,3‐triazol‐4‐yl)propane‐1,3‐diol backbone, two types of compounds (KARI 101 and KARI 201) each with alkyl groups of 10 or 9 chain lengths (Figure S1) exhibited potential effects as GHSR‐1a agonists [15]. This was confirmed by intracellular calcium influx assays, a measure of downstream GHSR‐1a signalling and activation, in HEK293A cells transiently transfected with the human GHSR‐1a [15]. To further validate these findings, functional assays were performed by Eurofins, an independent contract research organization. Consistent with our earlier results, both KARI 101 (EC_50_ = 1.85 ± 1.05 μM) and KARI 201 (EC_50_ = 3.36 ± 1.09 μM) elicited significant intracellular calcium responses in a dose‐dependent manner, reconfirming their agonistic activity toward GHSR‐1a (Figure 1A). Binding properties of KARI 101, KARI 201 and ghrelin (positive control) were assessed in competition binding assays using [^3^H]‐KARI 101 as the radioligand. Membrane preparations from human GHSR‐1a stable cells (Eurofins) were incubated with various concentrations of KARI 101, KARI 201 or ghrelin in the presence of [^3^H]‐KARI 101. The data showed a fit to the concentration–inhibition curve of these compounds, as observed in ghrelin treatment, and KARI 101, which has a longer alkyl chain, exhibited higher binding affinity than KARI 201 (Figure 1B).

To better understand the mechanism of KARIs/GHSR‐1a binding, we carried out molecular docking simulations. According to a previous structural study of ghrelin‐GHSR‐1a interactions, the residues Phe279 and Arg283 of transmembrane domain VI within GHSR‐1a were identified as key sites that interact most strongly with ghrelin, and these residues were shown to play a critical role in mediating intracellular calcium influx signalling [19, 20]. Docking simulations revealed that the triazole group of both KARI 101 and KARI 201 is positioned in close proximity to these critical residues, particularly Phe279 (Figure 1C). The numbers shown in the interaction map represent predicted interatomic distances between functional groups of the KARI compounds and amino acid residues within the GHSR‐1a binding pocket. Shorter interaction distances are indicative of stronger and more favourable noncovalent interactions, including hydrogen bonding, van der Waals contacts and alkyl interactions. Notably, KARI 101 exhibited shorter interaction distances with Phe279 (4.85) compared with KARI 201 (5.44), suggesting stronger and more stable interactions at the primary ghrelin‐binding pocket. This interpretation is further supported by the calculated docking scores (DS) and glide energy (GE) values, which were modestly more favourable for KARI 101 (DS = −1.767, GE = −37.395) than for KARI 201 (DS = −1.08, GE = −37.299). These structural predictions are consistent with the stronger calcium influx responses and higher binding affinity observed for KARI 101 in functional assays. Together, these results demonstrate that KARI 101 and KARI 201 act as potent agonists that directly interact with critical residues essential for GHSR‐1a signalling.

### 3.2 KARIs With Excellent Brain Penetration Promote Food Intake and Gastrointestinal Transit via Activation of ARC Neurons

**FIGURE 2:**
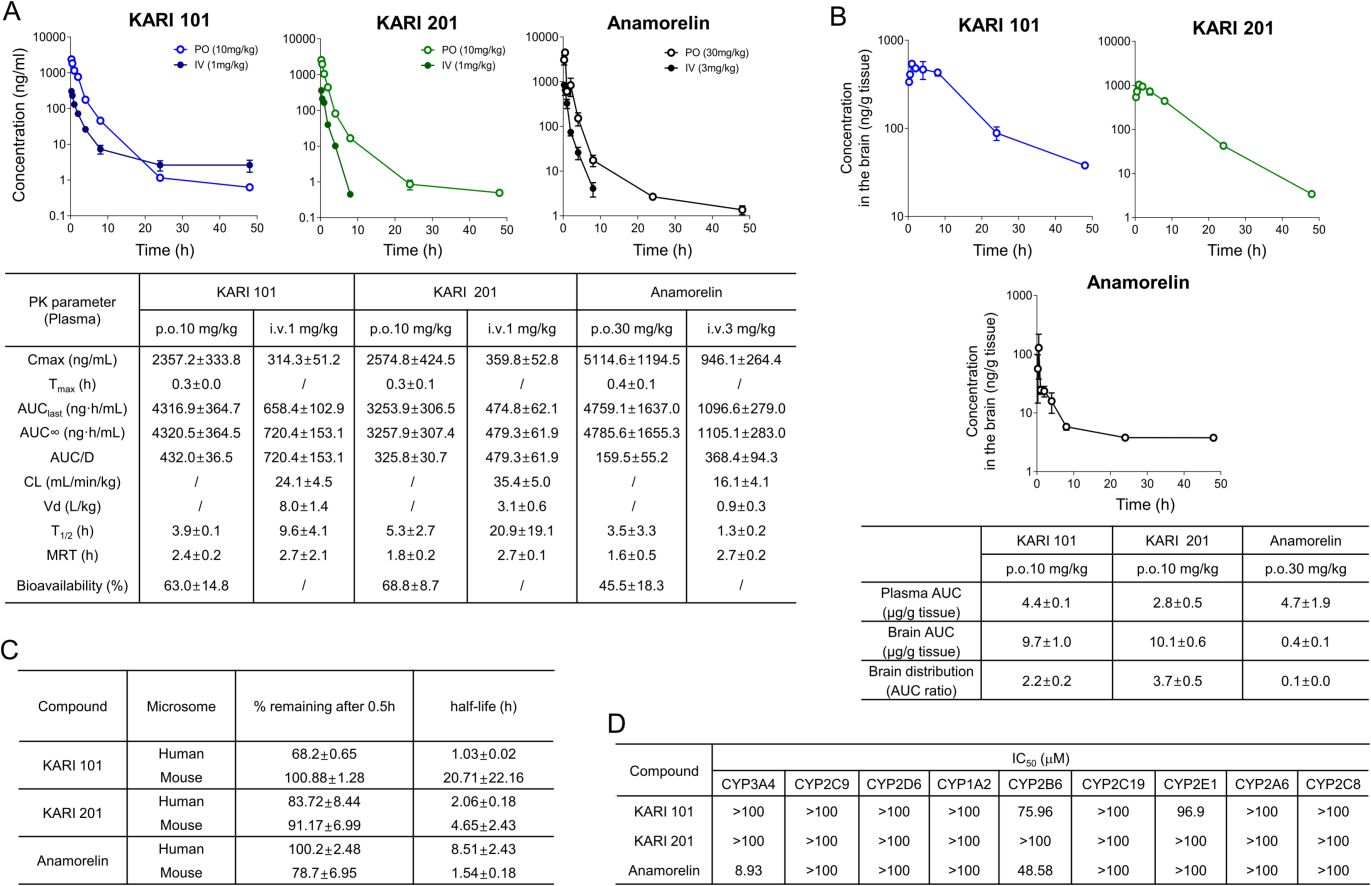
KARIs exhibit greater oral bioavailability and brain distribution compared with anamorelin. (A, B) Mean plasma (A) and brain (B) concentration vs. time profiles of KARI 101, KARI 201 and anamorelin after single dose p.o. (10 or 30 mg·kg^−1^) and i.v. (1 or 3 mg·kg^−1^) administration (plasma, *n* = 7 mice per group; brain, *n* = 4 mice per group). PK parameters of each compound in plasma (A) and brain (B) after p.o. or i.v. administration in mouse. (C) % compound remaining after 0.5 h and the half‐life of each compound after incubation with human and mouse liver microsomes (*n* = 3 independent experiments per group). (D) The IC_50_ (μM) of nine major CYP isozymes by each compound (*n* = 3 independent experiments per group). All error bars or data are mean ± SEM and may not be visually discernible in some cases due to their small magnitude.

Before investigating the biological effects of KARI compounds in vivo, we re‐examined their pharmacokinetic (PK) properties and compared them with those of anamorelin, the only GHSR‐1a agonist currently approved for clinical use, which is authorized in Japan for the treatment of cancer cachexia. Although the EC_50_ of anamorelin, reported as 0.74 nM [21], and its binding affinity confirmed by competition binding assays and docking simulations in our system were better than those of the KARI compounds (Figure S2), plasma PK profiling revealed that the dose‐normalized AUC values (AUC/D) of KARI 101 and KARI 201 were greater than those of anamorelin. Moreover, following oral administration, both compounds demonstrated greater systemic bioavailability (BA) compared with anamorelin (Figure 2A). Notably, the brain‐to‐plasma AUC ratios of KARI 101 and KARI 201 were significantly higher than that of anamorelin, indicating excellent brain penetration of the KARI compounds, whereas anamorelin exhibited poor brain distribution (Figure 2B). In metabolic stability assays using human and mouse liver microsomes, KARI 201 and anamorelin displayed high stability in human liver microsomes, whereas KARI 101 showed lower stability, consistent with our previous study [15] (Figure 2C). Evaluation of drug–drug interaction potential revealed that KARI compounds had no effect on the inhibition of the major nine cytochrome P450 enzymes, whereas anamorelin showed potential for CYP3A4 inhibition (Figure 2D). Therefore, these data confirmed the favourable PK properties of KARI 101 and KARI 201 as GHSR‐1a agonists, particularly their superior brain penetration compared with anamorelin.

**FIGURE 3:**
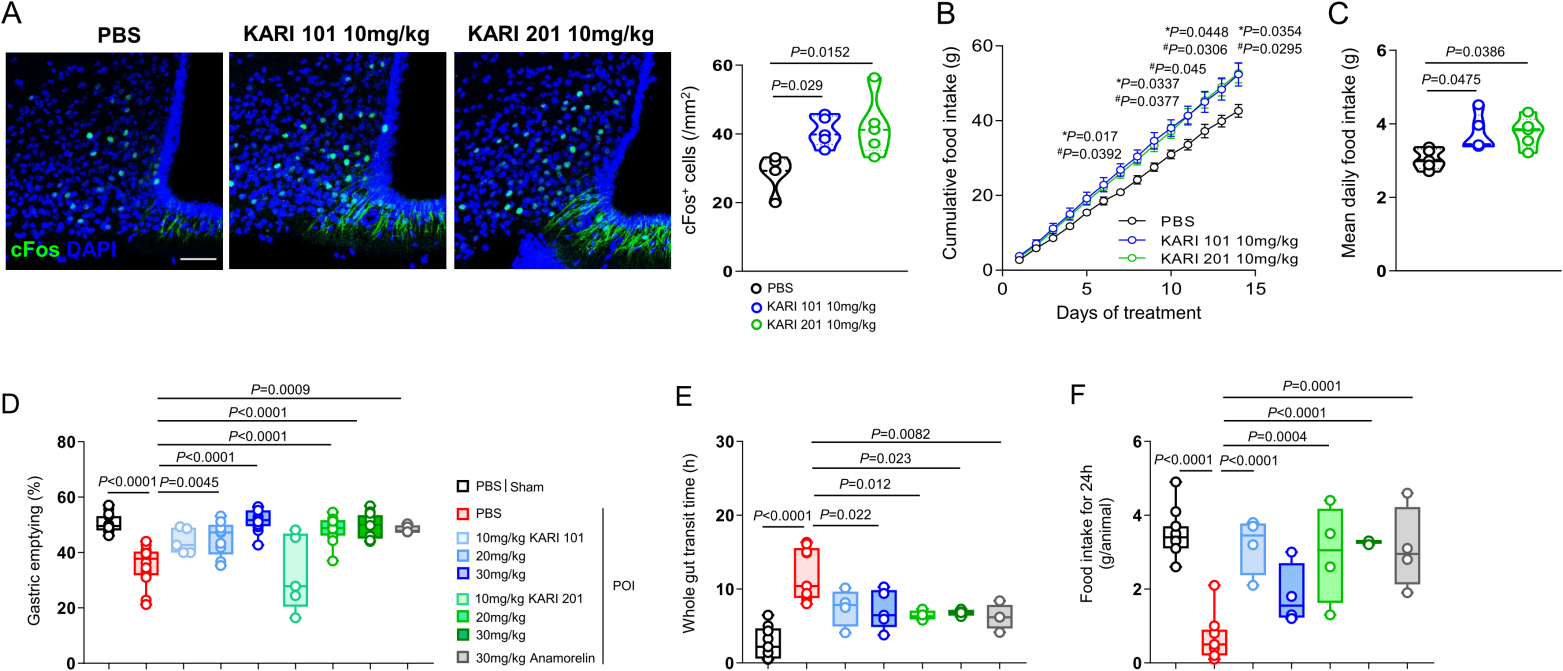
KARIs enhance food intake and gastrointestinal transit through activation of ARC neurons. (A) Representative images and quantification of c‐Fos^+^ cells (green) in ARC of C57BL/6 mice orally administered KARI 101 or KARI 201 once daily for 14 days (*n* = 5 mice per group). Scale bar, 50 μm. (B, C) Cumulative (B) and daily (C) food intake during each compound treatment period (*n* = 5 mice per group). * indicates significant differences compared with KARI 101, and # indicates significant differences compared with KARI 201. (D) Gastric emptying was evaluated by determining absorbance (A562) of phenol red in stomach homogenates collected 30 min after oral gavage of a methylcellulose–phenol red solution in POI mice treated with each compound (*n =* 5–15 mice per group). (E, F) Whole colonic transit time (E) and food intake (F) in POI mice treated with each compound (*n =* 4–11 mice per group). After 4‐h post POI surgery, randomly divided mice were administered orally PBS, KARI 101, KARI 201 or anamorelin, and each mouse was placed in clean metabolic cages for observation. The whole transit time until the first trypan blue‐stained faeces from the anus was measured. Food intake was measured 24 h after chemicals administration. One‐way analysis of variance, Tukey's post hoc test. All error bars indicate s.e.m.

To evaluate the GHSR‐1a agonistic effects of KARI compounds in vivo, we orally administered KARI 101 and KARI 201 at 10 mg·kg^−1^ daily for 2 weeks to C57BL/6 mice. These mice exhibited significantly increased neuronal activation in the hypothalamic arcuate nucleus (ARC), as measured by c‐Fos expression, which was accompanied by an increase in both cumulative and daily food intake (Figure 3A–C). Since ARC neuronal activation is known to enhance gastrointestinal motility via the vagus nerve [1, 2, 3, 4], we next examined the effects of KARI compounds on gastric emptying and colonic transit, in comparison with anamorelin, in a POI mouse model [16, 17]. Gastric emptying was markedly delayed in POI mice relative to nonsurgical controls but was improved by single oral administration of KARI compounds (20 or 30 mg·kg^−1^) or anamorelin (30 mg·kg^−1^) (Figure 3D). Colonic transit time was also significantly shortened in these treatment groups compared to nonsurgical controls, resulting in increased faecal pellet output and enhanced food intake (Figures 3E,F and S3). Importantly, these results demonstrated that KARI compounds exert efficacy at lower doses than anamorelin, suggesting that their high brain permeability confers potent in vivo activity as GHSR‐1a agonists.

### 3.3 KARIs Attenuate Age‐Related Decline in Skeletal Muscle Mass and Function in Mice

**FIGURE 4:**
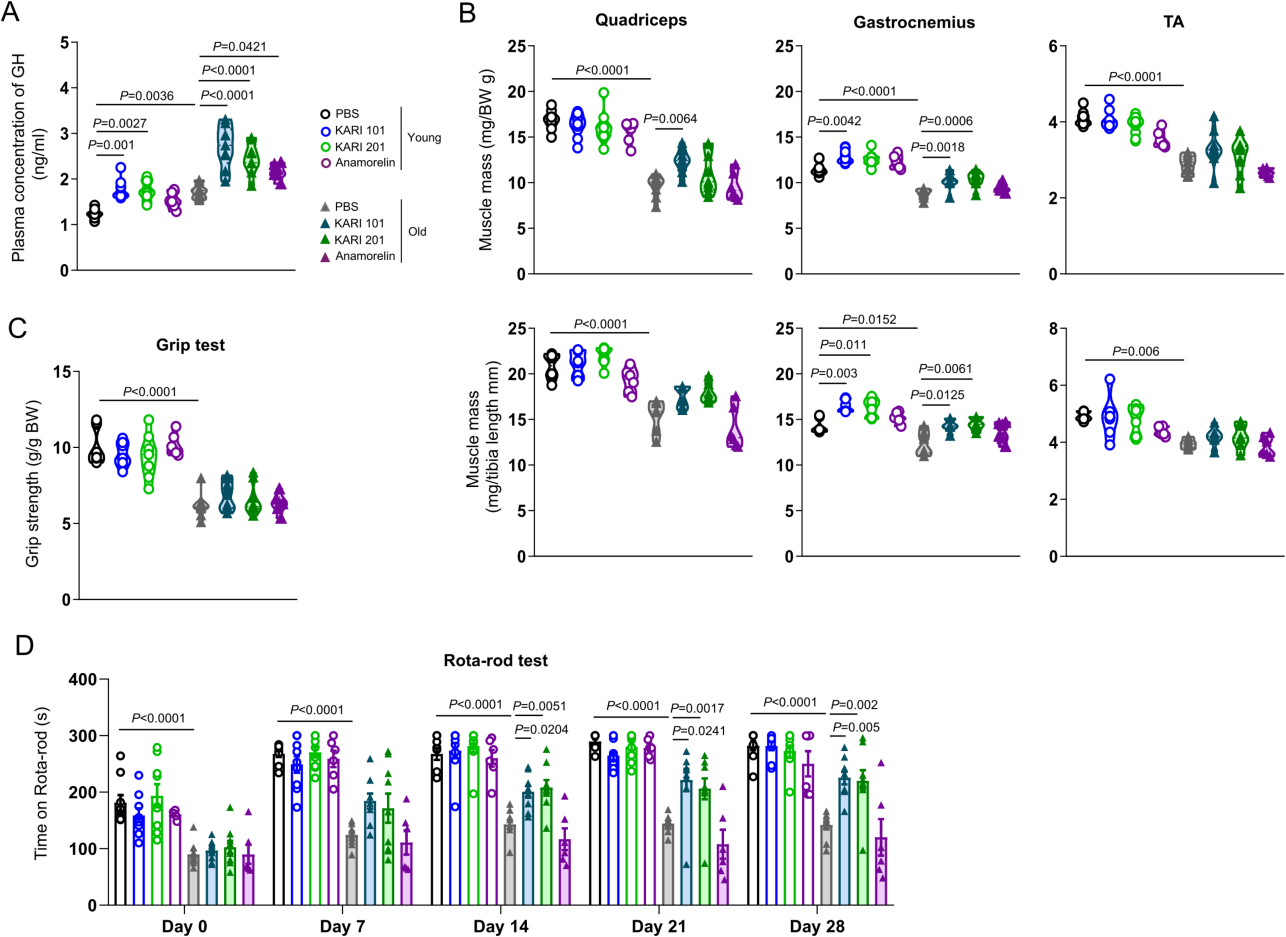
KARIs increase circulating GH levels and are associated with preservation of skeletal muscle mass and motor function in aged mice. (A) Plasma GH concentration in young (3 months old) and aged (22 months old) mice treated orally with KARIs (10 mg·kg^−1^) and anamorelin (30 mg·kg^−1^) once daily for 28 days (*n =* 6–9 mice per group). (B) Hindlimb muscles (quadriceps, gastrocnemius, and tibialis anterior (TA)) mass in each group, normalized to body weight (upper panels) or tibial length (lower panels) (*n =* 6–9 mice per group). (C) Grip strength measured on day 27 (*n =* 6–9 per group). (D) Rota‐rod performance assessed every 7 days (*n =* 6–9 per group). One‐way analysis of variance, Tukey's post hoc test. All error bars indicate s.e.m.

Activation of GHSR‐1a stimulates GH secretion from the pituitary gland, thereby contributing to peripheral effects such as adipose deposition and skeletal muscle growth [2, 4, 5]. To investigate whether KARI compounds exert these systemic actions, we orally administered 10 mg·kg^−1^ KARI 101 or KARI 201 daily for 4 weeks to both young (3 months old) and aged (23 months old) mice. For comparison, an anamorelin (30 mg·kg^−1^)‐treated group was included in both age groups. As expected, KARI compounds significantly increased plasma GH concentrations in these mice. Anamorelin treatment also elevated circulating GH levels in both age groups, although the magnitude of GH induction was lower than that observed with KARI compounds (Figure 4A). Despite this robust elevation of plasma GH, KARI‐treated mice did not exhibit significant changes in body weight or fat mass during the treatment period (Figure S4), suggesting that the primary anabolic effects of KARI compounds may not be directed toward adipose tissue. In contrast, anamorelin treatment resulted in a modest increase in body weight and fat mass in aged mice compared with PBS‐treated controls (Figure S4).

The effects on skeletal muscle were more pronounced. The reduced hindlimb muscle mass in aged mice, particularly in the gastrocnemius, was markedly increased by KARI 101 and KARI 201 treatment, and similar increases were also observed in young mice, but not by anamorelin (Figure 4B). Based on these results, we performed grip strength and rota‐rod test to evaluate muscular function. Grip strength measurements confirmed the expected age‐related decline in muscle force; this deficit was not significantly improved by either KARI compounds or anamorelin (Figure 4C). However, mice treated with KARI compounds exhibited a progressive improvement in rota‐rod performance, showing a trend toward improvement at Day 7 and reaching statistical significance at Days 14, 21 and 28, whereas anamorelin treatment did not result in significant improvement under the same conditions (Figure 4D). These results indicated that brain‐penetrant KARI compounds increase circulating GH levels to a greater extent than anamorelin and are associated with more robust preservation of skeletal muscle mass and improvements in motor performance in aged mice.

### 3.4 KARIs Impact Skeletal Muscle Protein Degradation and Synthesis of Aged Mice

**FIGURE 5:**
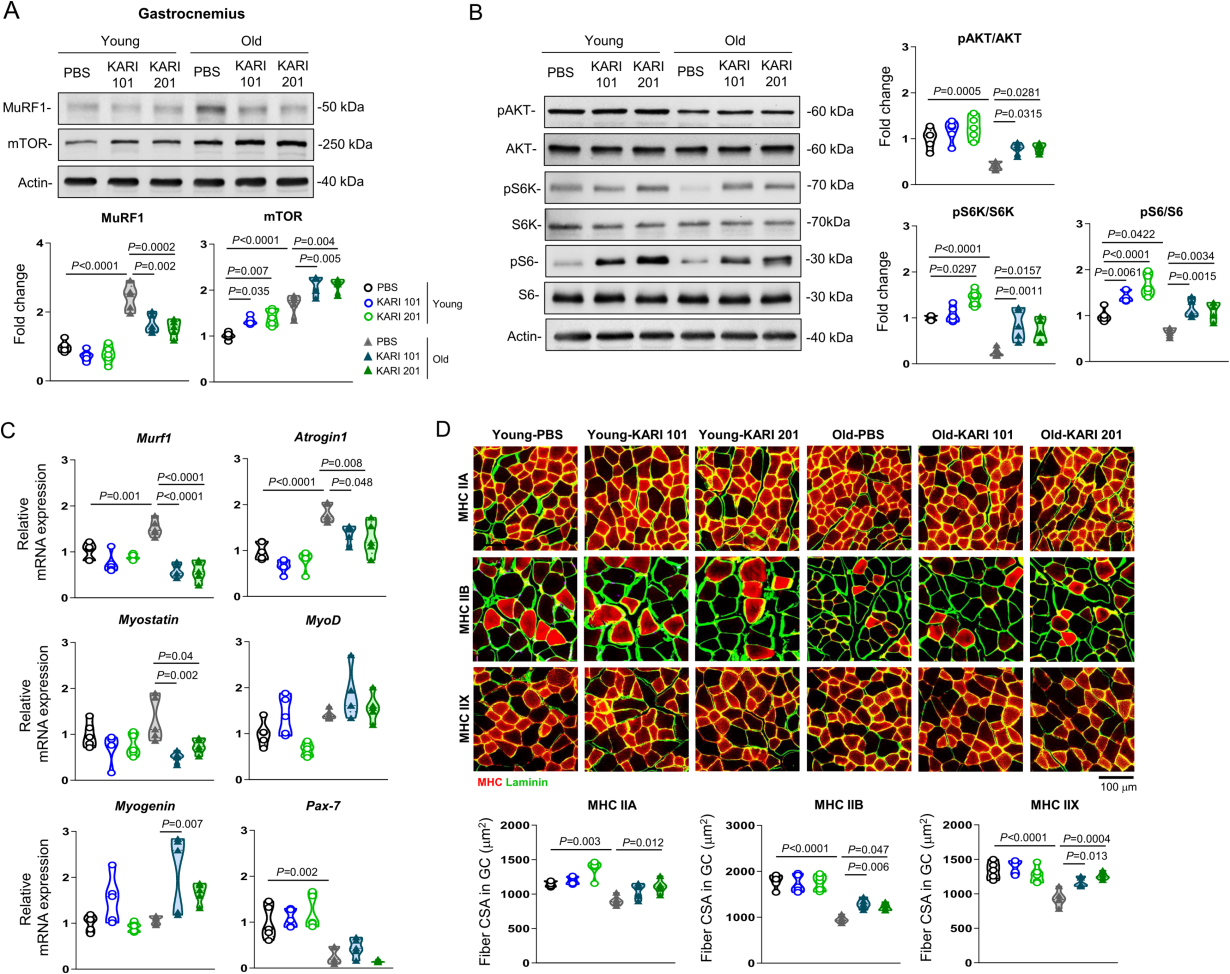
KARIs modulate skeletal muscle protein degradation and synthesis in aged mice. (A) Representative immunoblots and quantitative analyses of MuRF1 and mTOR in the gastrocnemius muscles from young (3 months old) and aged (23 months old) mice following treatment with each compound (*n =* 5 per group). (B) Representative immunoblots and quantitative analyses of phosphorylation and total protein levels of AKT, S6K, and S6 in the gastrocnemius muscles of each group (*n =* 5 mice per group). Phosphorylation levels were quantified as the ratio of phosphorylated to total protein from the same lane; all images were acquired under nonsaturating conditions. (C) mRNA expression of atrophic genes *(Murf1*, *Atrogin1* and *Myostatin*) and myogenic genes (*MyoD*, *Myogenin* and *Pax‐7*) in the gastrocnemius muscle of each group (*n =* 5 mice per group). (D) Representative images and muscle fibre cross‐sectional area (CSA) of immunohistochemistry MyHC staining for IIA (red), IIB (red) and IIX (red) with membranes stained for laminin (green) in the gastrocnemius muscle of each group (*n =* 5 mice per group). Scale bar, 100 μm. One‐way analysis of variance, Tukey's post hoc test. All error bars indicate s.e.m.

To further explore the pathways underlying the increase in skeletal muscle mass induced by KARI treatment in aged mice, we assessed protein expression related to muscle protein degradation and synthesis, including muscle RING‐finger protein‐1 (MuRF1) and mechanistic target of rapamycin (mTOR) [22, 23]. Aged mice exhibited elevated MuRF1 protein expression in gastrocnemius muscle compared with those of young mice, but this was markedly reduced by treatment with KARI 101 and KARI 201. In contrast, mTOR expression was notably increased in the gastrocnemius muscles of aged mice following KARI compounds administration (Figure 5A). Given that mTOR signalling is primarily regulated through posttranslational modifications [24], we next examined the activation status of key components of the AKT, S6K and S6 in the gastrocnemius muscle. Western blot analyses revealed that KARI treatment significantly increased the phosphorylation ratios of AKT, S6K and S6 in both groups, while total protein levels of AKT, S6K and S6 remained largely unchanged (Figure 5B). These findings indicate that KARI compounds activate mTOR signalling predominantly through posttranslational mechanisms rather than through changes in total protein abundance.

Consistent with these findings, we also observed significant downregulation of atrophic genes, including *Murf1*, *Atrogin1* and *Myostatin*, together with a trend toward upregulation of myogenic genes such as *MyoD* and *Myogenin* in gastrocnemius muscle of KARI compounds‐treated aged mice (Figure 5C). Moreover, we examined muscle size by immunostaining different muscle fibre types in gastrocnemius muscle. Aged mice showed decrease in cross‐sectional area (CSA) of Type IIA, IIB and IIX fibres compared with young mice, which was increased by KARI 101 and KARI 201 treatment (Figure 5D). Although the improvements in quadriceps and tibialis anterior (TA) muscles were less pronounced than those observed in the gastrocnemius, both muscles showed a consistent trend toward reduced atrophic signalling, enhanced mTOR pathway activation and increased muscle fibre size in KARI‐treated aged mice (Figures S5 and S6). Taken together, these results indicated that KARI compounds attenuate skeletal muscle loss by suppressing atrophic pathway and enhancing myogenic pathway in aged mice, suggesting their potential therapeutic effects in age‐related sarcopenia.

### 3.5 KARIs Improve Anorexia, Skeletal Muscle Loss and Functional Impairment in Cancer Cachexia Mice

**FIGURE 6:**
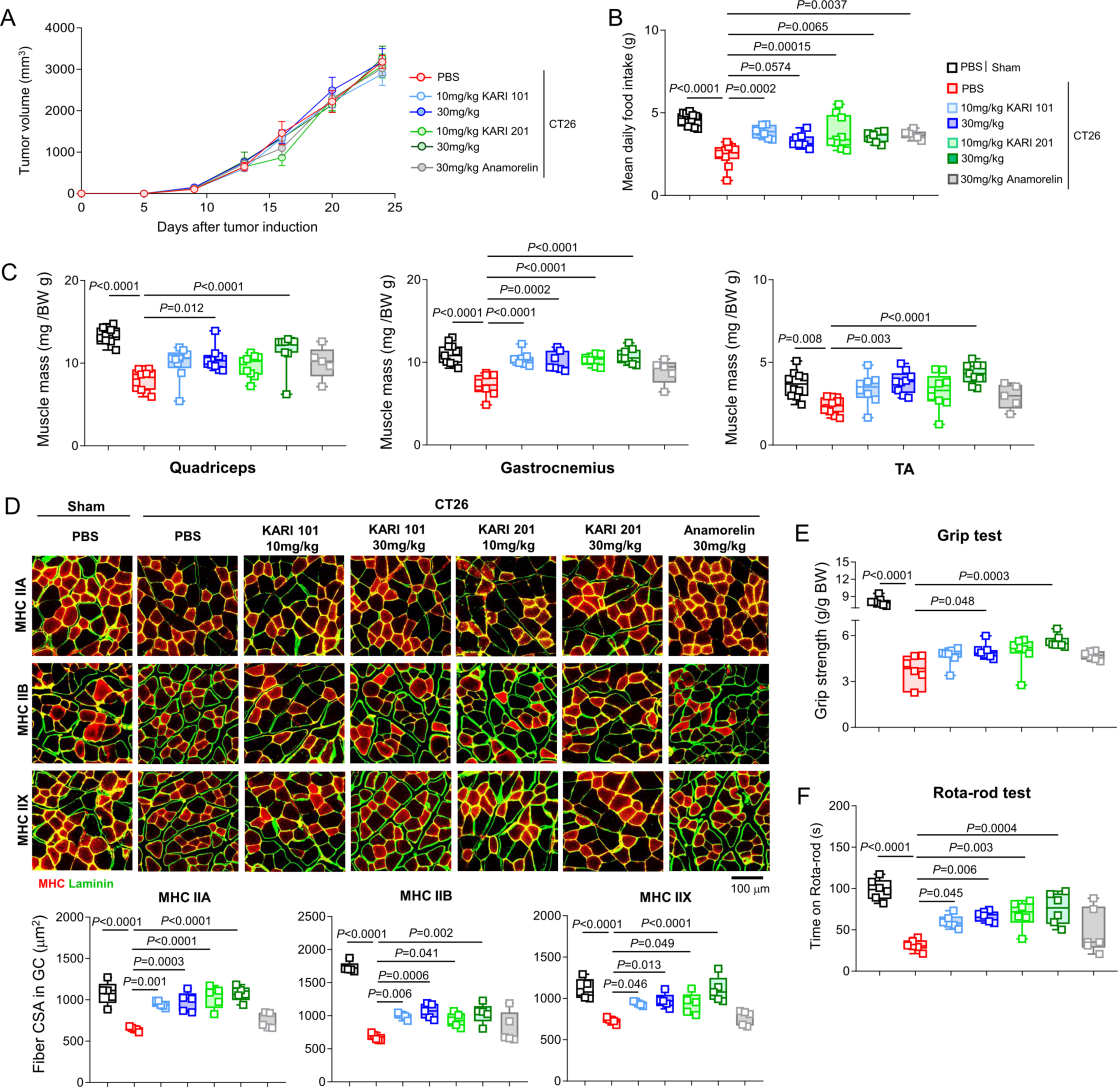
KARIs ameliorate anorexia, muscle wasting, and functional impairment in CT26‐bearing mice. (A) Tumour growth during the treatment period with each compound treatment. Tumour volumes were calculated using the formula: tumour volume (mm^3^) = 0.52 × length × width^2^ in which length (*n =* 6 mice per group). (B) Mean daily food intake measured every 4 days before mice were sacrificed (*n =* 6–13 mice per group). (C) Hindlimb muscles mass in each group (*n =* 5–13 mice per group). (D) Representative images and muscle fibre cross‐sectional area (CSA) of immunohistochemistry MyHC staining for IIA (red), IIB (red) and IIX (red) with membranes stained for laminin (green) in the gastrocnemius muscle of each group (*n =* 5 mice per group). Scale bar, 100 μm. (E, F) Grip strength (E) and rota‐rod (F) tests performed at day 22 and 23, respectively (*n =* 6–7 mice per group). One‐way analysis of variance, Tukey's post hoc test. All error bars indicate s.e.m.

Cancer cachexia is a multifactorial syndrome characterized by anorexia and the progressive wasting of skeletal muscle mass, ultimately leading to severe functional decline [25]. To investigate whether KARI compounds could mitigate these cachexia‐related features, we employed a well‐established mouse model induced by colon‐26 (CT26) tumour cells. Nine days after tumour inoculation, when tumours became measurable, mice were treated orally with PBS, KARI compounds (10 or 30 mg·kg^−1^) or anamorelin (30 mg·kg^−1^) daily until Day 24. As expected, CT26‐bearing mice treated with PBS exhibited substantial reductions in food intake, tumour‐free body weight and significant loss of fat and muscle mass compared with the nontumour bearing group. The administration of all doses of KARI compounds or anamorelin had no effect on tumour growth during the treatment period (Figure 6A). Moreover, despite their beneficial effects on food intake, neither KARI compounds nor anamorelin dramatically prevented the overall loss of body weight and fat mass in CT26‐bearing mice (Figures 6B and S7). In contrast, significant differences were observed in skeletal muscle outcomes. Treatment with KARI compounds at 30 mg·kg^−1^ markedly increased hindlimb muscle mass compared with the PBS group, whereas the same dose of anamorelin did not yield a significant improvement (Figure 6C). Notably, KARI compounds prevented gastrocnemius muscle loss at lower doses than anamorelin, highlighting their superior efficacy in preserving muscle mass (Figure 6C,D). Furthermore, grip strength was significantly enhanced at the 30 mg·kg^−1^ dose of KARI compounds, whereas rota‐rod performance was improved at both tested doses. In contrast, anamorelin did not produce measurable benefits in either test (Figure 6E,F). Collectively, these findings suggest that KARI compounds, through their GHSR‐1a agonistic activity in the brain, not only improve appetite but also preserve skeletal muscle mass and function, underscoring their promise as potential therapeutic agents for the treatment of cancer cachexia.

## 4 Discussion

In this study, we identified KARIs as potent small‐molecule GHSR‐1a agonists with favourable pharmacokinetic properties and excellent brain penetration, enabling them to modulate both central appetite and peripheral muscle growth and functional outcomes. These effects were observed consistently across multiple models, including wild type, postoperative ileus, age‐related sarcopenia and cancer cachexia, suggesting that KARIs may exert benefits in diverse physiological and pathological contexts. Unlike ghrelin and its analogues, which have long been studied as therapeutic candidates but remain limited by poor pharmacokinetics, short half‐life, enzymatic degradation and restricted brain permeability [10, 11], KARIs demonstrated favourable oral bioavailability and robust central exposure. While anamorelin represents an important clinical advancement as a small‐molecule GHSR‐1a agonist, our findings indicate that pharmacological differences among ghrelin receptor agonists can result in distinct physiological outcomes. In particular, although anamorelin increased circulating GH levels and body weight, this effect was not accompanied by consistent preservation of skeletal muscle mass or improvement in muscle function under our experimental conditions.

In this context, effective engagement of hypothalamic GHSR‐1a signalling may contribute to appetite regulation, whereas preservation of skeletal muscle mass and function likely involves coordinated endocrine and peripheral signalling. Specifically, the high brain penetrance of KARIs enables effective engagement of hypothalamic GHSR‐1a, leading to activation of central appetite‐regulating neuronal circuits, while concurrently enhancing GH release into the circulation, thereby contributing to peripheral anabolic effects on skeletal muscle. Rather than implying intrinsic superiority in receptor potency, our findings suggest that differences in pharmacokinetic and tissue distribution profiles may underlie the distinct functional outcomes observed among GHSR‐1a agonists. Therefore, our findings suggest that KARIs may represent a promising next‐generation approach to address key limitations of current ghrelin‐ and GHSR‐1a‐targeted therapies.

KARIs directly interacted with critical residues of GHSR‐1a and induced downstream calcium influx signalling. Notably, KARI 101, which has a longer alkyl chain, exhibited stronger calcium influx responses and binding affinity than KARI 201, suggesting that chain length contributes to receptor binding stability. This is consistent with previous studies showing that the octanoyl chain of ghrelin plays a critical role in receptor binding [20, 26]. Mechanistically, the activity of KARIs involves both central and peripheral actions of GHSR‐1a agonism. Centrally, KARI treatment activated ARC neurons, which enhanced food intake and promoted gastrointestinal transit via vagal pathways in the POI model, consistent with prior evidence that ARC activation regulates appetite and gastrointestinal motility [2, 3, 4]. Peripherally, ARC neuronal activation by KARIs increased circulating GH levels, which contributed to anabolic effects on skeletal muscle. These effects were associated with downregulation of atrophic factors such as MuRF1, together with upregulation of anabolic signalling pathways such as mTOR. Histological evidence of restored CSA in type IIB and IIX fibres further corroborated the muscle‐preserving effect of KARIs. Consequently, these compounds improved motor performance in models of age‐related sarcopenia and cancer cachexia. Importantly, these beneficial effects were observed at lower doses than anamorelin. Taken together, these findings suggest that the peripheral actions of brain‐penetrant GHSR‐1a agonism by KARIs extend beyond simple endocrine effects to confer structural and functional benefits in skeletal muscle.

Sarcopenia and cancer cachexia represent major clinical challenges, as they are associated with poor quality of life, impaired treatment tolerance and increased mortality. Despite the well‐recognized impact of anorexia and muscle loss in sarcopenia and cancer cachexia on patient outcomes, effective therapeutic options remain scarce. In particular, the most important features of cancer cachexia are a significant reduction in body weight and loss of skeletal muscle mass, with or without fat mass loss, indicating that muscle wasting represents the primary pathological hallmark of this syndrome [25, 27, 28]. Accordingly, targeting skeletal muscle preservation is crucial for achieving therapeutic efficacy. Anamorelin has demonstrated benefits in appetite stimulation in patients with cancer cachexia; however, its benefits remain suboptimal, particularly regarding muscle preservation and functional improvement [21, 29, 30]. By contrast, KARIs demonstrated superior brain penetration and efficacy in the promotion of food intake and restoration of muscle mass and motor performance, suggesting that they may address key unmet needs in the management of anorexia and muscular function for sarcopenia and cancer cachexia.

Nevertheless, there are several limitations in the current study. First, the relatively higher EC_50_ values of KARIs indicate that further optimization is needed to improve potency and specificity for GHSR‐1a. As noted, KARIs were originally developed as inhibitors of the sphingolipid enzyme acid sphingomyelinase (ASM), which is elevated in Alzheimer's disease [15]. Although we previously confirmed no off‐target effects of KARI 201 against 170 G protein‐coupled receptors, 430 kinases and 38 enzymes [15], we cannot fully exclude potential contributions from ASM inhibition that may have directly affected muscle outcomes. In this regard, previous studies have reported that excessive ASM activity and subsequent ceramide accumulation contribute to inflammatory signalling and muscle wasting [31, 32] and that ASM inhibition can ameliorate muscle inflammation and damage in conditions such as acute strenuous exercise, age‐related sarcopenia and cachexia [33, 34, 35, 36]. Thus, it is possible that the observed benefits of KARIs in muscle preservation may partly reflect combined effects of GHSR‐1a agonism and ASM inhibition. Second, our findings were derived from preclinical mouse models, and species‐specific differences in pharmacokinetics and receptor signalling may influence the translation of these results to humans. Third, while KARIs improved muscle mass and function, they had limited impact on fat mass or overall body weight, highlighting a selective action on muscle tissue that warrants further mechanistic study. Therefore, future investigations should include compound optimization, long‐term safety and efficacy assessments, evaluation in diverse sarcopenia and cachexia‐inducing tumour models and direct comparisons with other ghrelin analogues and GHSR‐1a agonists.

In conclusion, this study demonstrates that KARIs are potent, brain‐penetrant GHSR‐1a agonists with superior pharmacokinetic properties compared to anamorelin. By engaging both central and peripheral pathways, KARIs stimulate appetite, enhance gastrointestinal transit, increase GH secretion and preserve skeletal muscle mass and function. These multifaceted actions highlight their potential as promising therapeutic candidates for conditions characterized by anorexia and muscle wasting, including age‐related sarcopenia and cancer cachexia. Further development of KARIs‐based derivatives may provide a promising strategy to enhance therapeutic potential against conditions characterized by anorexia and muscle loss.

## Conflicts of Interest

The authors declare no conflicts of interest.

## Supporting information


**Figure S1:** Structure of KARI 101 (2‐amino‐2‐(1‐decyl‐1H‐1,2,3‐triazol‐4‐yl)propane‐1,3‐diol) and KARI 201 (2‐amino‐2‐(1‐nonyl‐1H‐1,2,3‐triazol‐4‐yl)propane‐1,3‐diol).
**Figure S2:** Binding affinity and molecular docking simulation of anamorelin. (A) Competition binding of anamorelin using 3H‐labelled KARI 101 as a radioligand. Averages of three independent experiments are shown. Data are mean ± SEM; n = 3 independent experiments. (B) Detailed interaction map through molecular docking simulation between ghrelin receptor (PDB ID:6KO5) and anamoreliln. Error bars represent s.e.m. and may not be visually discernible in some cases due to their small magnitude.
**Figure S3:** KARI compounds increase faecal pellet output in POI mice. (A, B) Faecal pellet number (A) and weight (B) in POI mice treated with each compound (n = 4–10 mice per group). After 4 h post POI surgery, randomly divided mice were administered orally PBS, KARI 101, KARI 201, or anamorelin and each mouse was placed in clean metabolic cages for observation. Faecal pellet out (pellet number and weight) was measured 24 h after chemicals administration. One‐way analysis of variance, Tukey's post hoc test. All error bars indicate s.e.m.
**Figure S4:** Effects of KARI compounds and anamorelin on body weight and fat mass in young and aged mice. (A, B) Body weight changes (A) during the treatment period and body weight gain (B) at days 14 and 28 in each group (n = 6–9 mice per group). (C) Subcutaneous and visceral fat mass in each group (n = 6–9 mice per group). One‐way analysis of variance, Tukey's post hoc test. All error bars indicate s.e.m.
**Figure S5:** KARI compounds are associated with preservation of quadriceps muscle characteristics in aged mice. (A) Representative immunoblots and quantitative analyses of MuRF1 and mTOR in the quadriceps muscles from young (3 months old) and aged (23 months old) mice following treatment with each compound (n = 5 per group). (B) Representative immunoblots and quantitative analyses of phosphorylated and total AKT, S6K and S6 in the quadriceps muscles of each group (n = 5 mice per group). Phosphorylation levels were quantified as the ratio of phosphorylated to total protein from the same lane; all images were acquired under nonsaturating conditions. (C) mRNA expression of atrophic genes (Murf1, Atrogin1 and Myostatin) and myogenic genes (MyoD, Myogenin and Pax‐7) in the quadriceps muscles of each group (n = 5 mice per group). (D) Representative images and muscle fibre cross‐sectional area (CSA) of immunohistochemistry MyHC staining for IIA (red), IIB (red) and IIX (red) with membranes stained for laminin (green) in the quadriceps muscles of each group (n = 5 mice per group). Scale bar, 100 m. One‐way analysis of variance, Tukey's post hoc test. All error bars indicate s.e.m.
**Figure S6:** KARI compounds are associated with preservation of tibialis anterior (TA) muscle characteristics in aged mice. (A) Representative immunoblots and quantitative analyses of MuRF1 and mTOR in the TA muscles from young (3 months old) and aged (23 months old) mice following treatment with each compound (n = 5 per group). (B) Representative immunoblots and quantitative analyses of phosphorylation and total protein levels of AKT, S6K and S6 in the TA muscles of each group (n = 5 mice per group). Phosphorylation levels were quantified as the ratio of phosphorylated to total protein from the same lane; all images were acquired under nonsaturating conditions. (C) mRNA expression of atrophic genes (Murf1, Atrogin1 and Myostatin) and myogenic genes (MyoD, Myogenin and Pax‐7) in the TA muscles of each group (n = 5 mice per group). (D) Representative images and muscle fibre cross‐sectional area (CSA) of immunohistochemistry MyHC staining for IIA (red), IIB (red) and IIX (red) with membranes stained for laminin (green) in the TA muscles of each group (n = 5 mice per group). Scale bar, 100 m. One‐way analysis of variance, Tukey's post hoc test. All error bars indicate s.e.m.
