# Supplementary material for: KARIs, Ghrelin Receptor Agonists With Excellent Brain Permeability, Increase Food Intake and Attenuate the Muscle Loss in Mice
Source: J Cachexia Sarcopenia Muscle. 2026 Apr 1;17(2):e70277. doi: 10.1002/jcsm.70277 (PMC13045456; doi:10.1002/jcsm.70277)
Supplement: Supplementary file 1 — Figure S1: Structure of KARI 101 (2‐amino‐2‐(1‐decyl‐1H‐1,2,3‐triazol‐4‐yl)propane‐1,3‐diol) and KARI 201 (2‐amino‐2‐(1‐nonyl‐1H‐1,2,3‐triazol‐4‐yl)propane‐1,3‐diol). Figure S2: Binding affinity and molecular docking simulation of anamorelin. (A) Competition binding of anamorelin using 3H‐labelled KARI 101 as a radioligand. Averages of three independent experiments are shown. Data are mean ± SEM; n = 3 independent experiments. (B) Detailed interaction map through molecular docking simulation between ghrelin receptor (PDB ID:6KO5) and anamoreliln. Error bars represent s.e.m. and may not be visually discernible in some cases due to their small magnitude. Figure S3: KARI compounds increase faecal pellet output in POI mice. (A, B) Faecal pellet number (A) and weight (B) in POI mice treated with each compound (n = 4–10 mice per group). After 4 h post POI surgery, randomly divided mice were administered orally PBS, KARI 101, KARI 201, or anamorelin and each mouse was placed in clean metabolic cages for observation. Faecal pellet out (pellet number and weight) was measured 24 h after chemicals administration. One‐way analysis of variance, Tukey's post hoc test. All error bars indicate s.e.m. Figure S4: Effects of KARI compounds and anamorelin on body weight and fat mass in young and aged mice. (A, B) Body weight changes (A) during the treatment period and body weight gain (B) at days 14 and 28 in each group (n = 6–9 mice per group). (C) Subcutaneous and visceral fat mass in each group (n = 6–9 mice per group). One‐way analysis of variance, Tukey's post hoc test. All error bars indicate s.e.m. Figure S5: KARI compounds are associated with preservation of quadriceps muscle characteristics in aged mice. (A) Representative immunoblots and quantitative analyses of MuRF1 and mTOR in the quadriceps muscles from young (3 months old) and aged (23 months old) mice following treatment with each compound (n = 5 per group). (B) Representative immunoblots and quantitative analyses of ph [file JCSM-17-e70277-s001.pdf]

Supplementary Information

KARIs, ghrelin receptor agonists with excellent brain permeability, increase food intake and attenuate the muscle loss in mice

1) **Supplementary materials and methods**
2) **Supplementary Figure S1-S8**
3) **Supplementary Table S1**

Supplementary materials and methods

1.1 Ligand binding assay

Ligand binding assays were performed using membrane preparations from GHSR-1a stable cell line (Eurofins, HTS187C). These cells were homogenized on ice in homogenization buffer containing 50 mM Tris-HCl (pH7.5), 5 mM EDTA, 5 mM $MgCl_2$, and EDTA-free protease inhibitor cocktail. The homogenate was ultracentrifuged at 100,000×g for 30 min at 4 °C, and the pellet was washed twice with cold homogenization buffer. The protein concentration was determined using BCA Protein Assay. All binding assays were conducted in reaction buffer containing 50 mM Tris-HCl (pH7.5), 5 mM EDTA, 5 mM $MgCl_2$, and 1% (w/v) bovine serum albumin. For the competition binding assay, 20 μg of membrane homogenates were incubated for 1 h at 37 °C with 0.001–10,000 nM ghrelin (Tocris, 1463), 0.01–100,000 nM KARI 101, KARI 201, or anamorelin (Sigma-Aldrich, SML2519) in the absence or presence of 1nM [^{3}H]-KARI 101 (RC Tritec). After incubation, the reaction was terminated by adding 100 μl of ice-cold reaction buffer containing 50 mM Tris-HCl (pH7.5), 5 mM EDTA, 5 mM $MgCl_2$, and 1% (w/v) bovine serum albumin and immediately filtered through P30 filtermat (Perkin-Elmer), followed by 5 times washing with ice-cold reaction buffer. Bound [^{3}H]-KARI 101 was quantified with using a Micro Beta 2 liquid scintillation counter (Perkin-Elmer). Data were analyzed by nonlinear curve-fitting using the program GraphPad Prism 8. Binding data are reported as means ± s.e.m.

1.2 Molecular docking

Molecular modeling studies were performed using Discovery Studio Programs (2018, Accelrys) and figures were generated using PyMOL programs. The 3D coordinates of human ASM were obtained from the protein data bank for docking studies (PDB entry 6KO5). Preparation of the protein was performed with the Prepare Protein protocol and a binding site was created using the Define and Edit Binding Site protocol (default parameters were used). Compounds were drawn in ChemDraw. Energy minimization of ligands was performed by the Clean Geometry module in Discovery Studio, and conformations were generated using the Generate Conformations protocol and the Best method. Analysis of ligand

interactions at the binding site was performed using the Ligand Interactions tool followed by visual inspection.

1.3 Microsomal stability

KARI 101, KARI 201 or anamorelin stock solutions (10 mM dissolved in methanol) were diluted with potassium phosphate buffer (pH7.4) to make a concentration of 10 μM. A 10 μl aliquot of this solution was then added to an 80 μl aliquot of 100 mM potassium phosphate buffer (pH7.4) containing human or mouse liver microsomes (80 μl of 0.625 mg protein ml^{-1} phosphate buffer) and incubated at 37 °C for 10 min before adding 10 μl of an NADPH regenerating system to start the reaction [15]. Reactions were stopped at 0, 15, 30, 60, and 90 min time-points by the addition of 200 μl cold acetonitrile (containing propranolol as internal standard at 20 ng ml^{-1}) and centrifuged at 13,200 rpm for 10 min. A 2 μl aliquot of the supernatant was injected into the LC-MS/MS system to analyze the remained concentrations of KARI 101, KARI 201 or anamorelin. The ratio of the peak area of the test compound/internal standard was used to determine the % remaining of each compound over time. The half- life ($T_{1/2}$) and % compound remaining after 30 min were then calculated.

1.4 Cytochrome P450 inhibition

KARI 101, KARI 201 or anamorelin stock solutions (10 mM dissolved in methanol) were serially diluted with methanol to make concentrations of 100, 250, 500, 2500, and 10000 μM and then further diluted 5-fold using potassium phosphate buffer (pH7.4) [15]. Probe substrate cocktail solutions for seven major cytochrome P450 isozymes (phenacetin (100 μM for 1A2), bupropion (50 μM for 2B6), diclofenac (20 μM for 2C9), mephenytoin (100 μM for 2C19), dextromethorphan (5 μM for 2D6), chlorzoxazone (50 μM for 2E1), midazolam (5 μM for 3A4), coumarin (20 μM for 2A6), and paclitaxel (20 μM for 2C8) were prepared with phosphate buffer. A 5 μl aliquot of each compound solution and a 5 μl aliquot of each probe substrate cocktail solution was incubated at 37 °C with human liver microsomes (80 μl of 0.625 mg protein ml^{-1} phosphate buffer) for 10 min before the addition of 10 μl of the NADPH regenerating system to start the reaction. Reactions were stopped at 15 min by the addition of 200 μl cold acetonitrile (containing propranolol as

internal standard at 20 ng ml^{-1}) and centrifuged at 14,000 rpm for 10 min. A 2 μl aliquot of the supernatant was injected into the LC-MS/MS system [15]. The ratio of the peak area of the probe substrate/internal standard was used to determine the % metabolic activity of probe substrate according to the concentrations of KARI 101, KARI 201 or anamorelin added.

1.5 Gastric emptying

After 20 h post POI surgery, mice were randomly assigned to the blank, PBS, KARI 101 (10, 20, 30 mg·kg^{-1}), KARI 201 (10, 20, 30 mg·kg^{-1}), or anamorelin (30 mg·kg^{-1}) treatment group. The PBS or each compound were orally treated to the mice. After 4 h, all group were orally administered a 1.5 % methylcellulose solution containing 0.05 % phenol red at 0.2 ml/mouse. Mice were killed 30 min after injection at which time the entire stomach was immediately isolated. For baseline control, the blank group was killed immediately to confirm the volume of methylcellulose detected in the stomach at time zero. The stomach and its contents were homogenized with 4 ml of 0.1 N NaOH. An additional volume of 6 ml of 0.1 N NaOH was added and after centrifugation at 3,000 rpm for 10 min at 4 °C, an aliquot of 1 ml of the supernatant was removed and mixed with 100 μl of 20 % trichloroacetic acid. After another centrifugation at 3,000 rpm, 500 μl of the supernatant was mixed with 400 μl of 0.5 N NaOH and the absorption measured at 562 nm (A 562). The percentage of gastric emptying was determined as: (1-[A 562 of test sample/A 562 of baseline control]) × 100.

1.6 Evaluation of colonic transit time and fecal pellet output

After 4h post POI surgery, randomly divided mice were administered orally PBS, KARI 101, KARI 201, or anamorelin and each mouse was placed in clean metabolic cages for observation. The whole transit time until the first trypan-blue-stained feces from the anus was measured [17]. Fecal pellet out (pellet number and weight) and food intake was measured 24 h after chemicals administration.

1.7 Immunofluorescence staining

For immunofluorescence staining, brain was cut on a vibratome (30 μm). The c-Fos (mouse, 1:100, Abcam, ab208942) antibody was used to confirm neuronal activity in the hypothalamus. Alexa anti-mouse 488 was used as secondary antibodies. The brain sections were analyzed with a laser-scanning confocal microscope (FV3000; Olympus) or with a BX51 microscope (Olympus). MetaMorph software (Molecular Devices) was used for quantification. For muscle staining, fresh gastrocnemius muscle from the right hindlimb of each animal were mounted with OCT and flash-frozen in isopentane-chilled liquid nitrogen. Each muscle tissue OCT block was coronally sectioned at 10 μm at -25°C on a Leica CM3050S Cryostat (Leica, Nussloch, Germany). Sections were dried at room temperature (RT) for 30 minutes, then blocked in 10% goat serum for 1 hour. Slides were incubated in primary antibodies for 2 hours at RT. Primary antibodies were as follows: SC-71 (Myosin heavy chain, IIA, mouse, 1:100), BF-F3 (Myosin heavy chain, IIB, mouse, 1:100), 6H1 (Myosin heavy chain, IIX, mouse, 1:50) all from Developmental Studies Hybridoma Bank, Iowa City, IA, and laminin (rabbit, 1:500, PA1-16730, Invitrogen). Alexa anti-mouse 594 and Alexa anti-rabbit 488 were used as secondary antibodies. Slides were imaged at 20x and a complete cross-sectional image was obtained by slide scanner (SLIDEVIEW VS200; Olympus). To quantify the cross-sectional areas (CSA) in the gastrocnemius muscle, blinded trained researchers used ImageJ Fiji analysis software. The representative images were obtained using a laser-scanning confocal microscope (FV3000; Olympus).

1.8 Western blot analysis

Samples were lysed in RIPA buffer (Cell Signaling Technology, 9806), then subjected to SDS-PAGE and transferred to a nitrocellulose membrane. Membranes were blocked with 5 % BSA, incubated with primary antibody and then incubated with the appropriate horseradish peroxidase-conjugated secondary antibody [15]. Primary antibodies to the following proteins were used: MuRF1 (1:500, Santa cruz, sc-398608), mTOR (1:1000, Cell Signaling Technology, 2983), pAKT (1:1000, Cell Signaling Technology, 4060), AKT (1:1000, Cell Signaling Technology, 4691), pS6K (1:500, Cell Signaling Technology, 9234), S6K (1:500, Cell Signaling Technology, 2708), pS6 (1:1000, Cell Signaling Technology, 2215), S6 (1:1000, Cell Signaling Technology, 2217), and β-Actin

(1:1000, Santa cruz, sc-47778). Mouse-HRP (1:1000, Cell Signaling Technology, 7076) and Rabbit-HRP (1:1000, Cell Signaling Technology, 7074) were used as secondary antibody. We performed densitometric quantification using the ImageJ software (National Institutes of Health). Images have been cropped for presentation and uncropped immunoblots are provided in Figure S8.

1.9 ELISA

For measurement of mouse growth hormone, mouse blood was collected into sodium heparin-coated tubes via intracardial bleed at the time of death. Plasma was generated by centrifugation of freshly collected blood and aliquots were stored at -80 °C until use. We used commercially available ELISA kits (NOVUS, MBP3-08143). ELISA was then performed according to the manufacturer's instructions.

1.10 RNA isolation and real-time PCR analysis

RNA was extracted from the brain homogenates and cell lysates using the RNeasy Lipid Tissue Mini kit and RNeasy Plus Mini kit (QIAGEN) according to the manufacturer's instructions. cDNA was synthesized from 5 μg of total RNA using a commercially available kit (Takara Bio Inc.). Quantitative real-time PCR was performed using the Thermal Cycler Dice Real Time System III (Takara). Primer sequences are listed in Table S1.

Supplementary Figures

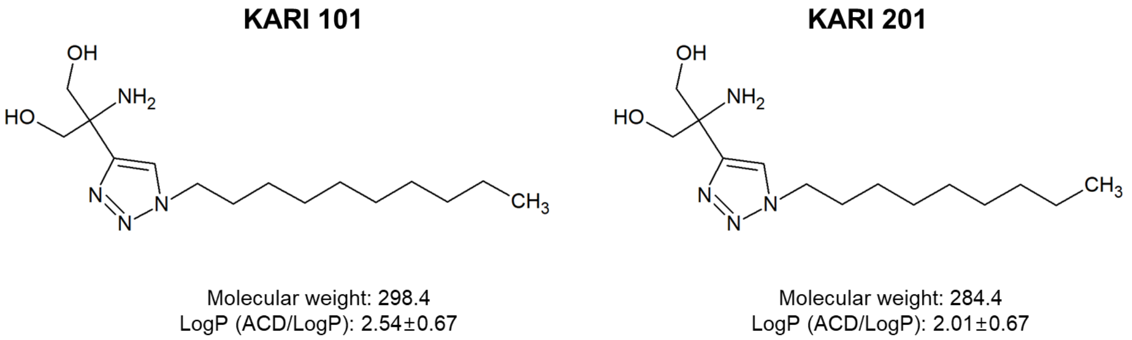

Figure S1. Structure of KARI 101 (2-amino-2-(1-decyl-1H-1,2,3-triazol-4-yl)propane-1,3-diol) and KARI 201 (2-amino-2-(1-nonyl-1H-1,2,3-triazol-4-yl)propane-1,3-diol).

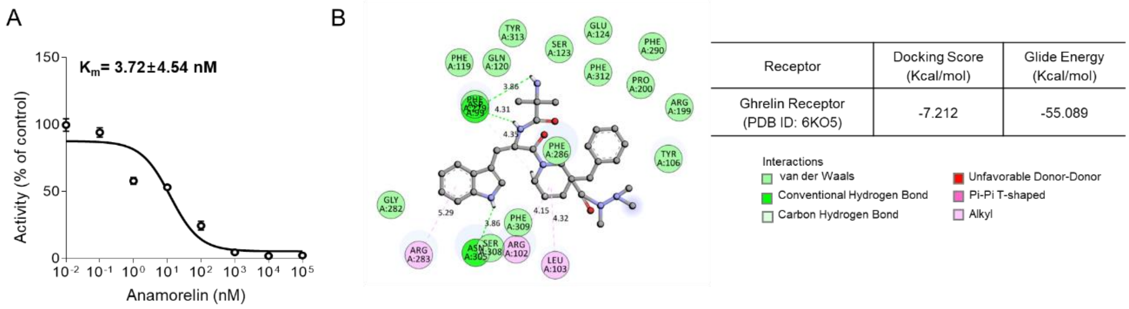

Receptor	Docking Score (Kcal/mol)	Glide Energy (Kcal/mol)
Ghrelin Receptor (PDB ID: 6KO5)	-7.212	-55.089

Figure S2. Binding affinity and molecular docking simulation of anamorelin. (A) Competition binding of anamorelin using ^{3}H-labeled KARI 101 as a radioligand. Averages of three independent experiments are shown. Data are mean ± s.e.m.; n = 3 independent experiments. (B) Detailed interaction map through molecular docking simulation between ghrelin receptor (PDB ID:6KO5) and anamoreliln. Error bars represent s.e.m. and may not be visually discernible in some cases due to their small magnitude.

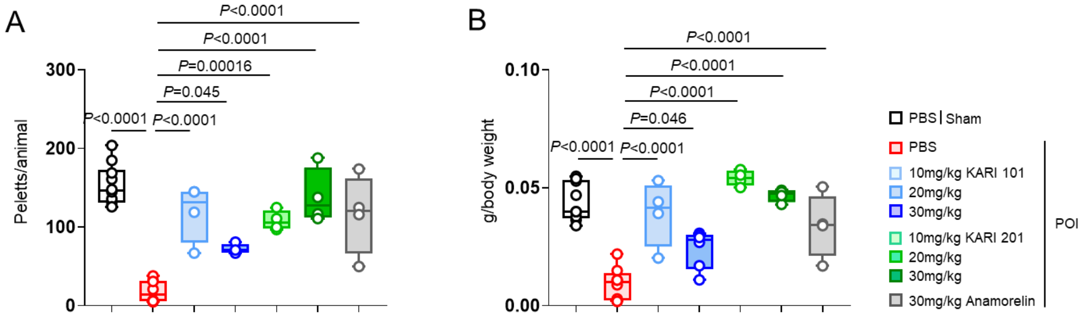

Figure S3. KARI compounds increase fecal pellet output in POI mice. (A, B) Fecal pellet number (A) and weight (B) in POI mice treated with each compound (n = 4-10 mice per group). After 4h post POI surgery, randomly divided mice were administered orally PBS, KARI 101, KARI 201, or anamorelin and each mouse was placed in clean metabolic cages for observation. Fecal pellet out (pellet number and weight) was measured 24 h after chemicals administration. One-way analysis of variance, Tukey's post hoc test. All error bars indicate s.e.m.

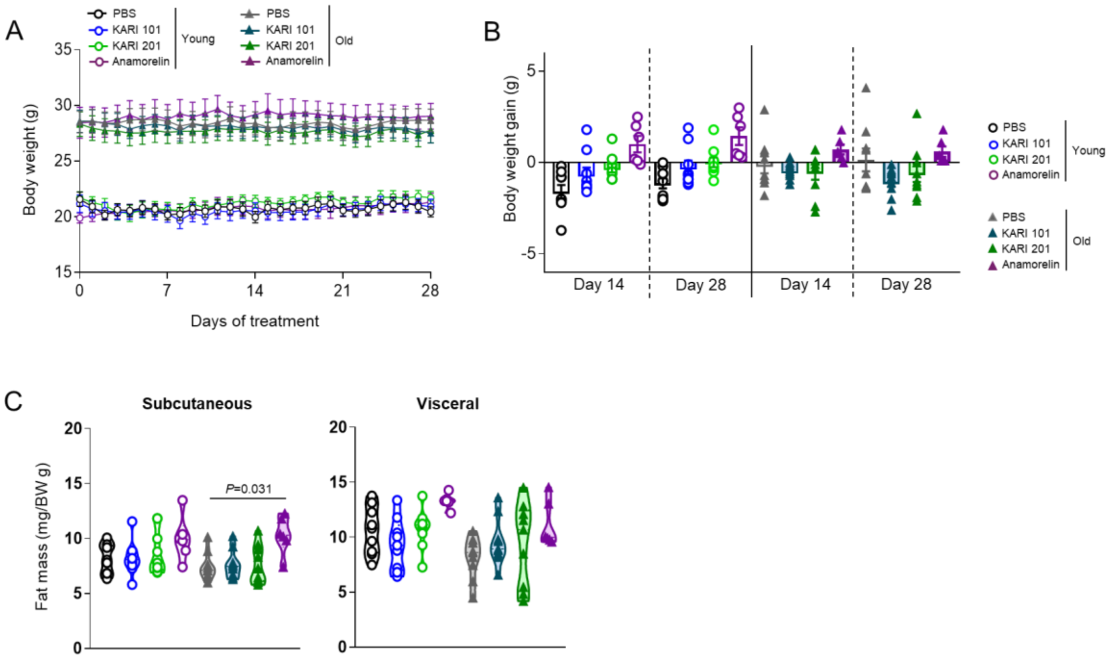

Figure S4. Effects of KARI compounds and anamorelin on body weight and fat mass in young and aged mice. (A, B) Body weight changes (A) during the treatment period and body weight gain (B) at days 14 and 28 in each group (n = 6-9 mice per group). (C) Subcutaneous and visceral fat mass in each group (n = 6-9 mice per group). One-way analysis of variance, Tukey’s post hoc test. All error bars indicate s.e.m.

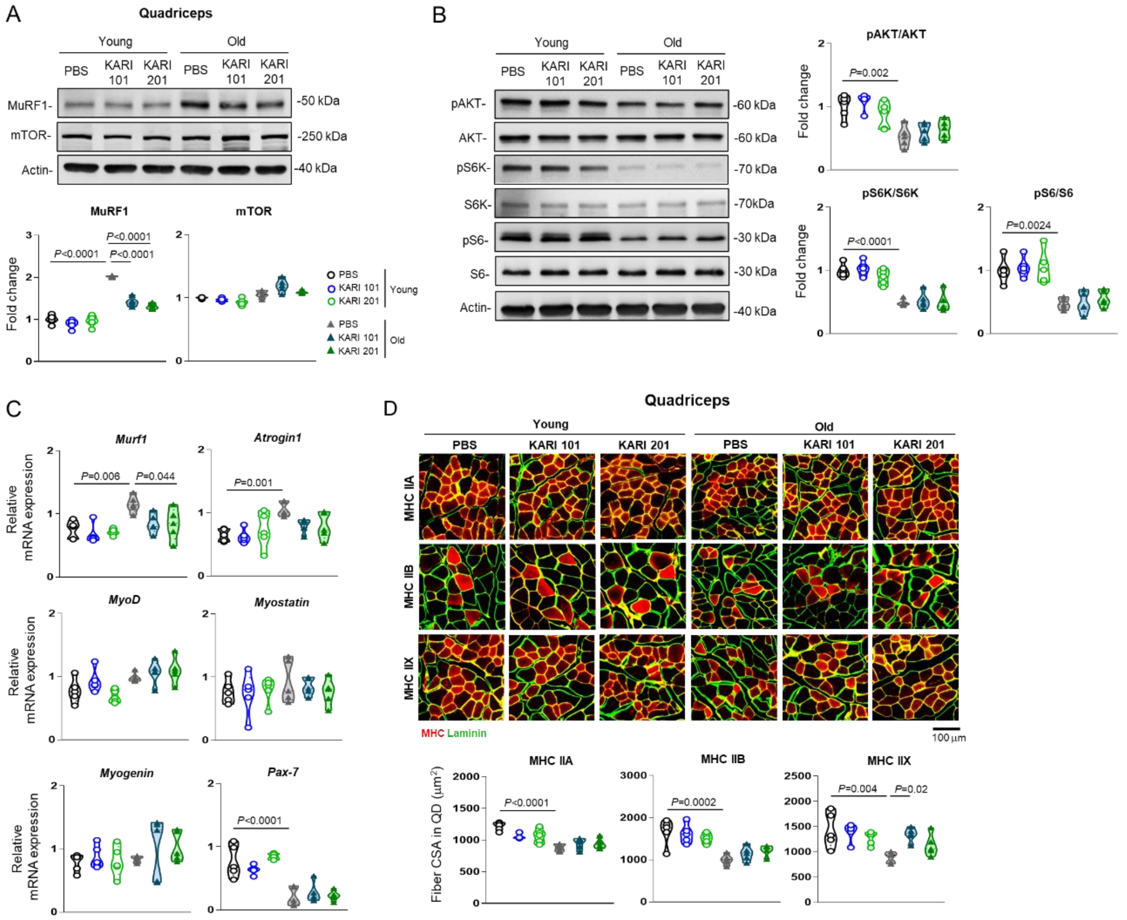

Figure S5. KARI compounds are associated with preservation of quadriceps muscle characteristics in aged mice. (A) Representative immunoblots and quantitative analyses of MuRF1 and mTOR in the quadriceps muscles from young (3-month-old) and aged (23-month-old) mice following treatment with each compound (n = 5 per group). (B) Representative immunoblots and quantitative analyses of phosphorylated and total AKT, S6K, and S6 in the quadriceps muscles of each group (n = 5 mice per group). Phosphorylation levels were quantified as the ratio of phosphorylated to total protein from the same lane; all images were acquired under non-saturating conditions. (C) mRNA expression of atrophic genes (*Murf1*, *Atrogin1*, and *Myostatin*) and myogenic genes (*MyoD*, *Myogenin*, and *Pax-7*) in the quadriceps muscles of each group (n = 5 mice per group). (D) Representative images and muscle fiber cross-sectional area (CSA) of

immunohistochemistry MyHC staining for IIA (red), IIB (red), and IIX (red) with membranes stained for laminin (green) in the quadriceps muscles of each group (n = 5 mice per group). Scale bar, 100 μm. One-way analysis of variance, Tukey's post hoc test. All error bars indicate s.e.m.

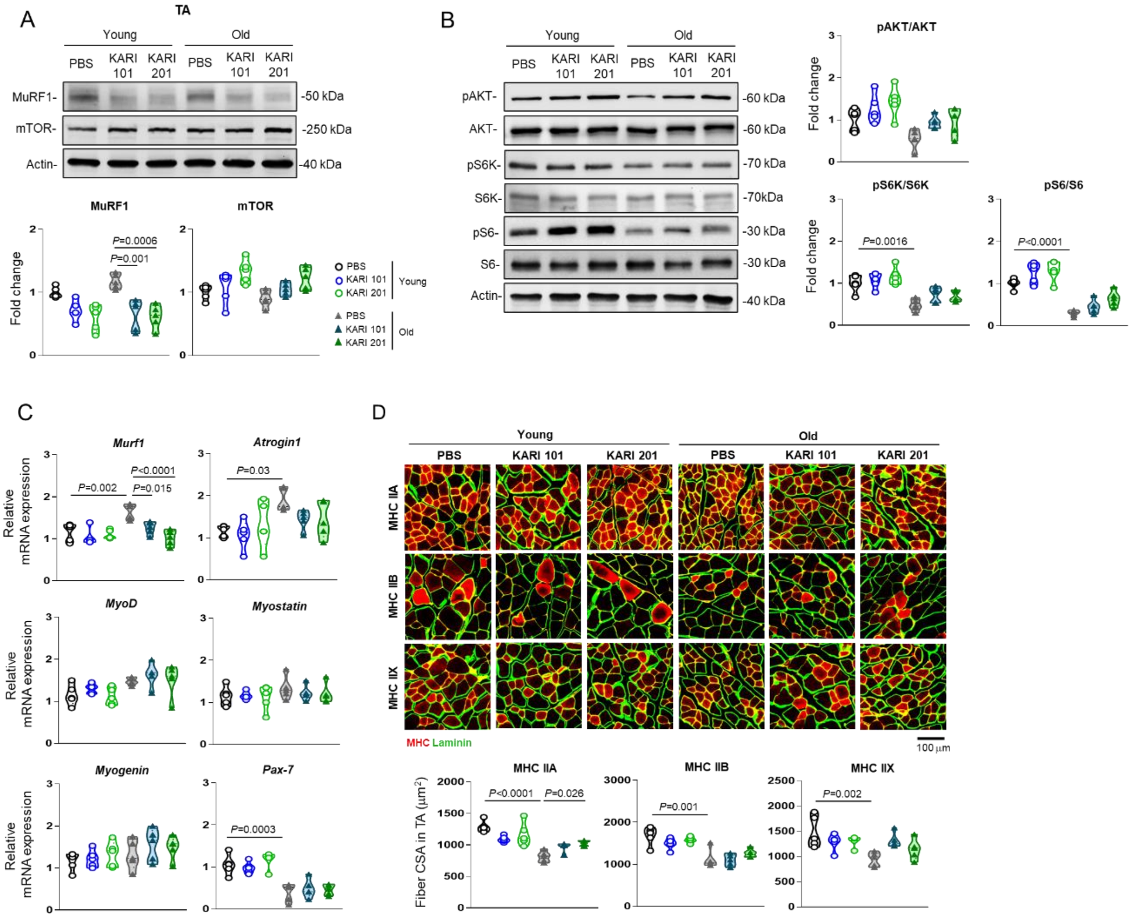

Figure S6. KARI compounds are associated with preservation of tibialis anterior (TA) muscle characteristics in aged mice. (A) Representative immunoblots and quantitative analyses of MuRF1 and mTOR in the TA muscles from young (3-month-old) and aged (23-month-old) mice following treatment with each compound (n = 5 per group). (B) Representative immunoblots and quantitative analyses of phosphorylation and total protein levels of AKT, S6K, and S6 in the TA muscles of each group (n = 5 mice per group). Phosphorylation levels were quantified as the ratio of phosphorylated to total protein from the same lane; all images were acquired under non-saturating conditions. (C) mRNA expression of atrophic genes (*Murf1*, *Atrogin1*, and *Myostatin*) and myogenic genes (*MyoD*, *Myogenin*, and *Pax-7*) in the TA muscles of each group (n = 5 mice per group). (D) Representative images and muscle fiber cross-sectional area (CSA) of

immunohistochemistry MyHC staining for IIA (red), IIB (red), and IIX (red) with membranes stained for laminin (green) in the TA muscles of each group (n = 5 mice per group). Scale bar, 100 μm. One-way analysis of variance, Tukey's post hoc test. All error bars indicate s.e.m.

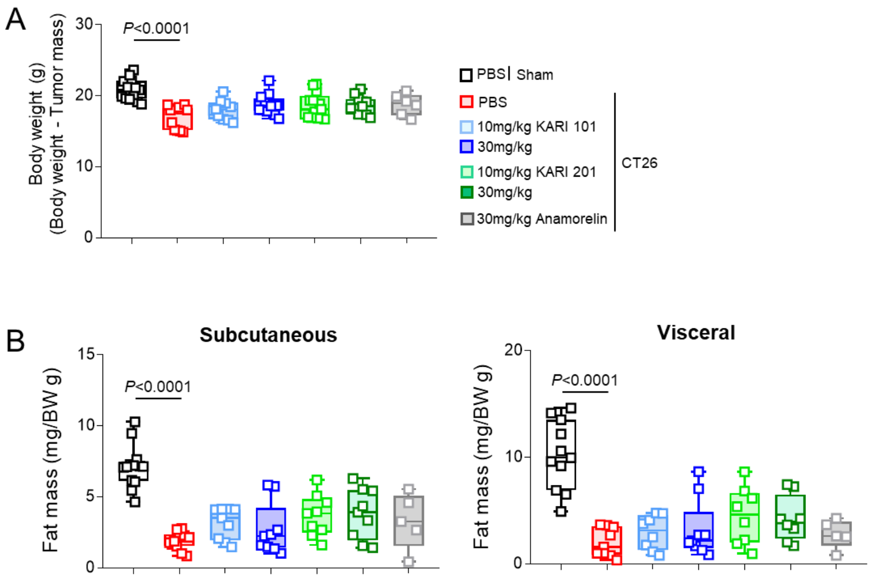

Figure S7. KARI compounds did not affect body weight and fat mass in CT26-bearing mice. (A) Tumor-free body weight before mice were sacrificed (n = 6-13 mice per group). (B) Subcutaneous and visceral fat mass in each group (n = 5-13 mice per group).One-way analysis of variance, Tukey's post hoc test. All error bars indicate s.e.m.

Figure S8. Uncropped western blots.

Figure 5A and 5B.

Boxes indicate regions shown in the figure.

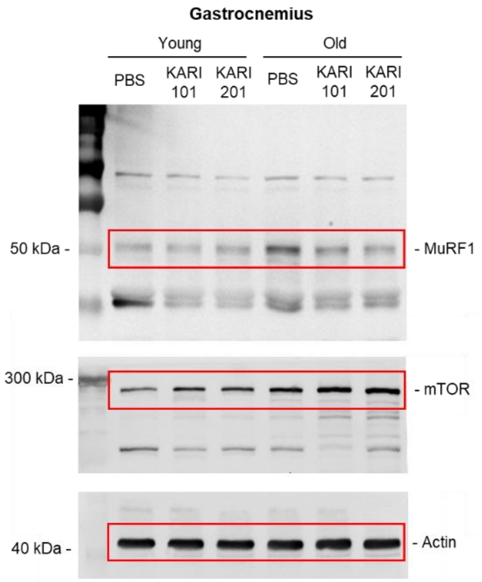

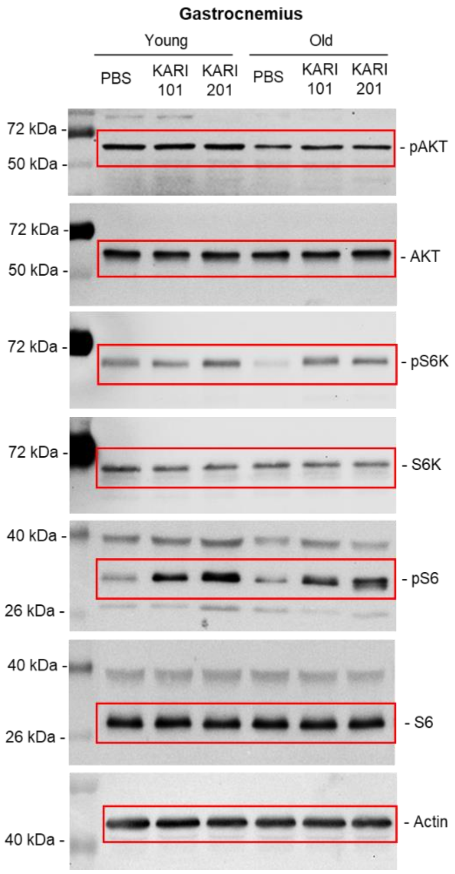

Figure S5A and S5B.

Boxes indicate regions shown in the figure.

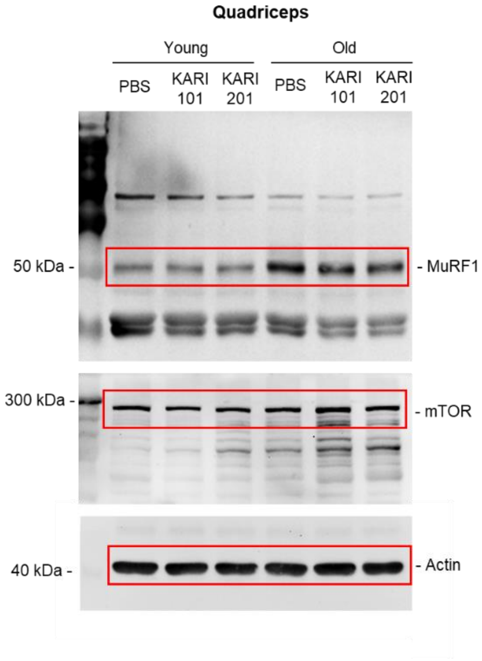

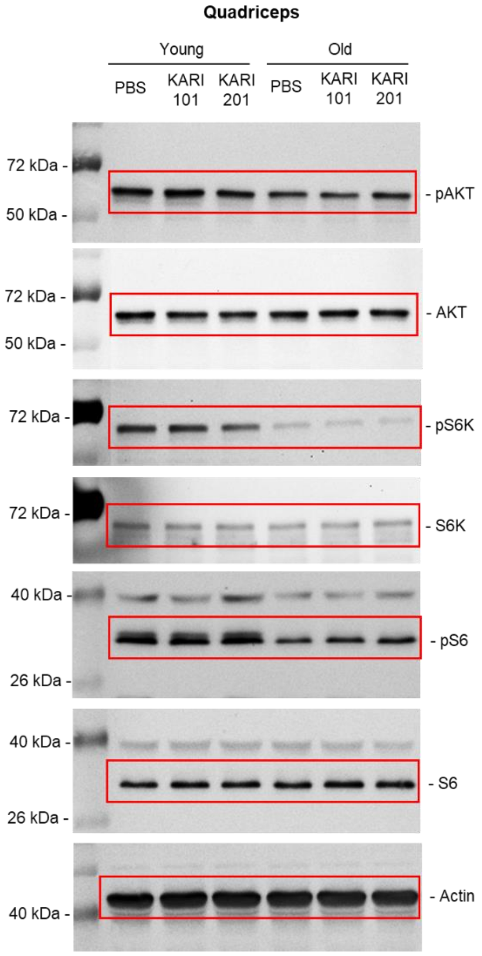

Figure S6A and S6B.

Boxes indicate regions shown in the figure.

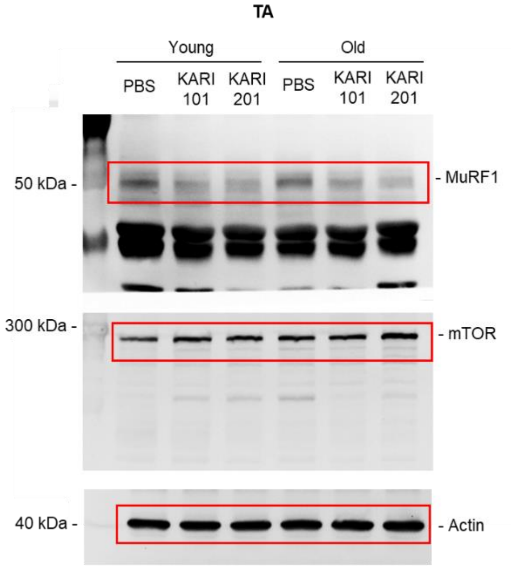

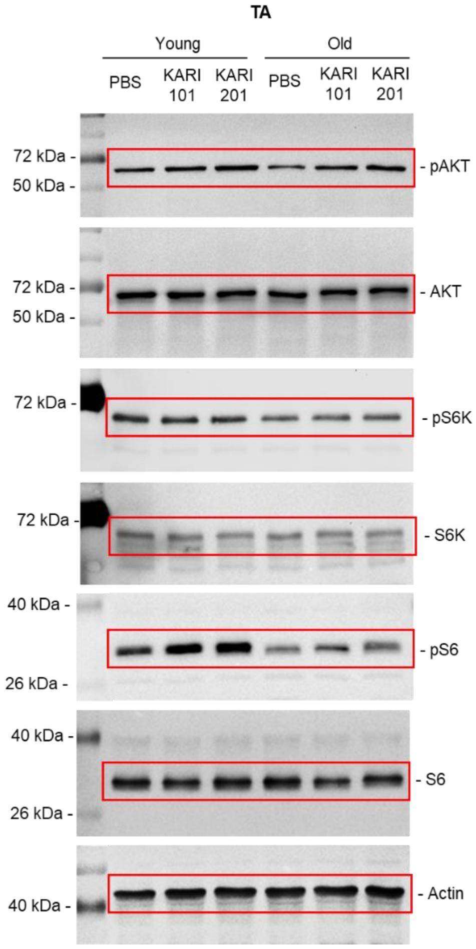

Supplementary Table

Table S1. Sequences of Real-time PCR primer pairs

Gene	Forward	Reverse
mMurf1	5’-CAACCTGTGCCGCAAGTG-3’	5’-CAACCTCGTGCCTACAAGATG-3’
mAtrogin1	5’-GCAGAGAGTCGGCAAGTC-3’	5’-CAGGTCGGTGATCGTGAG-3’
mMyostatin	5’-AGTGGATCTAAATGAGGGCAGT-3’	5’-GTTTCCAGGCGCAGCTTAC-3’
mMyoD	5’-CCACTCCGGGACATAGACTTG-3’	5’-AAAAGCGCAGGTCTGGTGAG-3’
mMyogenin	5’-GAGACATCCCCCTATTTCTACCA-3’	5’-GCTCAGTCCGCTCATAGCC-3’
mPax-7	5’-TCTCCAAGATTCTGTGCCGAT-3’	5’-CGGGGTTCTCTCTCTTATACTCC-3’
mGapdh	5’-TGAATACGGCTACAGCAACA-3’	5’-AGGCCCCTCCTGTTATTATG-3’

Murf1, muscle RING-finger protein-1; *MyoD*, myoblast determination protein; *Pax-7*, Paired box 7; *Gap dh*, glyceraldehyde 3-phosphate dehydrogenase.
